# Annotations

**Published:** 1896-05-23

**Authors:** 


### Annotations.

### Some Medical Aspects of Education.

We desire to call attention to an address delivered to the members of the Folkestone branch of the National Educational Union by Dr. Percy Lewis of that town. Dr. Lewis says little that is not familiar to the medical man, but his pamphlet is throughout a kind of protest against the want of intelligence which prevails among those who are placed in charge of the education and upbringing of boys and girls. Here, for example, is an elementary fact which ought to be at the fingers' ends of every school teacher who has passed the age of fifteen; and yet it may be questioned whether nine in every ten even of the head masters of our public schools have any but the very faintest comprehension of its real significance. Dr. Lewis is speaking of the value of properly regulated muscular exercises, and he tells us that "the muscles, which have been called the furnaces of the body, have been estimated to contribute about four-fifths of the heat necessary to maintain life. . . . Children with badly-developed muscles have feeble circulations, cold hands and feet, and feeble brains." No doubt in our public schools the general exercises are such as to keep the strong in a fair condition of muscular activity and development; but what about many of our girls' schools? Above all, what about both the boys and girls who are cooped up, and have relatively no playgrounds, in the board schools of our larger towns? The subject is all-important, and is as successfully neglected as it can well be. Let us hope that Dr. Lewis and those who think with him will enter upon a much more energetic propaganda than they appear to have yet contemplated.

### A Fruit Cure for Alcoholism.

The Honourable Dudley Campbell is one of the newest of our social regenerators. His methods aim at nothing less than the entire extirpation of disease, and every other form of unhappiness, root and branch. It is an excellent intention, and we wish Mr. Campbell all imaginable success. It cannot be said that he has yet quite accomplished everything which he says his principles and panaceas are capable of accomplishing. If he had, we should not venture to offer a single word of criticism. But inasmuch as, in spite of Mr. Campbell's panaceas, there are still some small ills left in the world, we will venture to submit to some simple tests his short and easy methods of putting an end to all human ills. Drunkenness, it will be admitted, is a very serious social evil. Now this evil, which many philanthropists and physicians of large and weighty experience have essayed to cure, presents no difficulties to the Honourable Dudley Campbell. "Are you a chronic drunkard?" says Mr. Campbell, in effect. "Why, then, hey presto! eat plenty of fruit, and the finest champagne in the world will never tempt you again." Listen to his words, incredulous man of science, and doubt no more! "Hitherto," says this experienced regenerator, "hitherto temperance advocates have, in the main, been content with devices for mitigating the ravages of drink, leaving untouched the cause." Foolish temperance advocates! "Thus," he continues, "they insist on the duty of self-control," &c. . . . "But were the cause eradicated, such devices would be needless; and this might be done by a way as simple as the washing in the river Jordan recommended to the leper captain by Elisha, namely, a free use of fruits at every meal. That the desire for alcoholic beverages would thus cease is proved by thousands of examples." Thousands of examples ought to be very convincing. We venture to propose a practical test. It is this. Let him go and find ten—not ten thousand—but ten confirmed drunkards of long standing anywhere in Great Britain, and try his "fruits at every meal" upon them. If he cures two out of the ten he will write a much less rhetorical pamphlet than his "Fruits and Gardens for the People." But it will also be much more practical and convincing. Fruit is good, very good indeed in its place; but rhetoric in place of solid scientific fact is mostly rubbish.

### Why is the Negro Black?

It has occurred to a writer in an American medical newspaper to discuss the question of the blackness of the negro's skin. It will be a revelation to many to learn that the baby negro is not born black. Even so long ago as 1765 Le Cat noticed that the newly-born negro is of a reddish colour. That observation has since been frequently confirmed; and it is now pretty widely known that, though the baby negro begins to follow in the footsteps of his parents as regards colour within a few days after birth, yet at the moment of birth he shows a disposition to aspire towards the civilised races, being white, or, at worst, red in hue. It is generally assumed that the primest of all the causes of a negro's blackness is the hot sun beneath whose more or less vertical rays he is doomed to live. There is, however, a physiological condition of the skin which differentiates that organ from the integument of an European. "The negro," says our American scientist, "possesses a more developed vascular sudoriparous system than we do." In other words, he has more and larger sweat glands, and they are more liberally supplied with blood. By means of these he perspires much more abundantly. This condition is possibly a contributory factor in his blackness. It is an important element in the investigation to remember, however, that the blackest of all black people are almost invariably found under certain very definite climatic conditions. That is to say, they are found where great heat, strong light, and much atmospheric moisture are in combination. For example, "the blackest negroes in Africa are those who live in Guinea where the greatest amount of rain annually falls." On the other hand, "the people who live in the dry section of the Nubian Desert have red skins." Heat, light, and humidity are all causes of pigmentation, and if to these we add the fact of the highly "developed vascular sudoriparous system" of the negro, we have travelled as far as our American investigator is able to help us. The question is one of genuine scientific interest; and, perhaps, when the Matabele, and the Dervishes, and the Soudanese have all settled down quietly in the ways of civilisation and order, science may turn her attention in this direction, and tell us much that is both new and interesting about those races who differ so markedly from ourselves in colour, character, and many other particulars.

